# Recurrent Meningothelial Meningioma With Multiple Extensions: A Complex Case Study

**DOI:** 10.7759/cureus.50826

**Published:** 2023-12-20

**Authors:** Corneliu Toader, Razvan-Adrian Covache-Busuioc, Bogdan-Gabriel Bratu, Luca-Andrei Glavan, Matei Serban, Alexandru Vladimir Ciurea

**Affiliations:** 1 Department of Neurosurgery, Carol Davila University of Medicine and Pharmacy, Bucharest, ROU; 2 Department of Vascular Neurosurgery, National Institute of Neurology and Neurovascular Diseases, Bucharest, ROU; 3 Department of Neurosurgery, Sanador Clinical Hospital, Bucharest, ROU

**Keywords:** radiotherapy, tumor recurrence, surgical resection, seizure management, ataxic gait, neurological complications, intracranial neoplasms, meningothelial meningioma

## Abstract

This case report presents a comprehensive analysis of a 67-year-old patient diagnosed in 2017 with meningothelial meningioma, focusing on the challenges of managing such tumors and their neurological implications. Meningiomas, being the most common benign intracranial neoplasms, have a notable research gap regarding their association with seizures and motor deficits. This patient, who had a history of depressive disorder, persistent cephalalgia syndrome, and ataxic gait, initially presented with symptoms including ataxic gait, confusion, and headache. Imaging revealed a large, hyperdense right frontal meningioma with a significant mass effect. Following surgical resection, the patient experienced notable neurological improvement. However, in 2023, the patient re-presented with bradypsychia, bradykinesia, and memory disorders, indicating a recurrent meningioma. This case exemplifies the recurrence and complex management of meningiomas, particularly in elderly patients, and highlights the importance of individualized treatment strategies. Surgical resection remains the primary treatment approach, supplemented by radiotherapy in cases of recurrence or incomplete resection. The case underscores the need for advancements in therapeutic approaches to mitigate recurrence risks and enhance patient outcomes in meningioma management. This is especially pertinent given the tumor's predilection for older females and its varied neurological manifestations, such as ataxic gait and seizures.

## Introduction

Meningiomas, recognized as the most prevalent benign intracranial neoplasms, exhibit a notable deficiency in research regarding the incidence and determinants of seizure occurrences in affected patients. This gap is particularly evident when contrasted with studies on other types of brain tumors. The development of enhanced methodologies for anticipating, managing, and inhibiting seizures in meningioma patients is a critical objective. This urgency is underlined by the substantial influence of tumor-induced epilepsy on patients' quality of life [1]. Seizures manifest as a primary symptom in 13-60% of intracranial meningioma cases, with surgical excision achieving total seizure control in 53-90% of patients who exhibited preoperative seizures [2]. Motor deficits, either focal or nonfocal, may arise in these patients. Focal deficits, such as hemiparesis, hemiplegia with spasticity, gait impairments, ataxia, and incoordination, are directly correlated with the anatomical regions impacted by the tumor or its treatment. Such deficits can occur in any tumor type, whether primary or secondary, but they are more frequently associated with primary malignant tumors [3]. The significant role of the cerebellum in gait regulation is evidenced by the ataxic gait characteristics observed in patients with cerebellar damage, where increased gait variability and step width are commonly linked to gait stability [4]. Supratentorial tumors, located in the cerebral hemispheres, predominantly affect younger patients. These include astrocytomas, gangliogliomas, craniopharyngiomas, supratentorial primitive neuroectodermal tumors, germ cell tumors, dysembryoplastic neuroepithelial tumors, oligodendrogliomas, and meningiomas [5]. Among grade I meningiomas, there are nine histologic subtypes: meningothelial, psammomatous, microcystic, fibrous, lymphoplasmacyte-rich, transitional, secretory, metaplastic, and angiomatous. Meningothelial meningiomas are characterized by their distinctive morphology, including whorls and streams of meningothelial cells with eosinophilic cytoplasm and indistinct cytoplasmic borders. They typically exhibit ovoid nuclei with nuclear grooves, clearing, and pseudoinclusions [6]. These tumors feature lobulated, nested, or whorled aggregates of cells, often surrounded by collagen or fibrous septae. They may also show nuclear atypia, which does not necessarily correlate with prognosis [7]. There is a notable gender disparity in the prevalence of meningiomas, with a significantly higher incidence in adult females compared to adult males. Approximately 90% of meningiomas are intracranial, while the remaining 10% occur in the spinal meninges. These tumors are predominantly observed in the elderly, with incidence rates increasing in individuals aged over 65 years [8]. Women experience more than a twofold higher incidence rate than men. Research suggests that sex hormones and genetic differences between genders may explain this disparity. Additionally, correlations have been observed between meningiomas and female hormone-associated conditions, such as breast cancer, uterine fibroids, and endometriosis [9].

## Case presentation

A 67-year-old patient, known to have a depressive disorder, persistent cephalalgia syndrome, and gait disorders (gait apraxia), has been experiencing symptoms since 2017. During the neurological examination, the patient exhibited an uncharacteristic attitude and gait apraxia, with staggering movements. Motor function and coordination were normal. Cutaneous reflexes showed bilateral flexion of the plantar type, osteo-tendinous reflexes presented bilaterally symmetrical, and as sensitivity, there was a headache, sphincter was continent, normal clinical relations of cranial nerves, the patient was confused, and Glasgow Coma Scale (GCS) was 14 points. A cerebral CT examination performed in 2017 revealed a voluminous right frontal expansive process. This process had a hyperdense, heterogeneous aspect with meningeal implantation, calcifications, hyperostosis of the calvarium, and a supraorbital position. Its overall dimensions were approximately 6/4 cm, with central necrosis and peripheral digitiform edema. There was a mass effect on the ventricular system, and the median axis was displaced to the left by 12 mm. A brain MRI examination at the time captured an inhomogeneous, right superior frontal extra-axial space-occupying tumor. It exhibited hyper/isointense T2 and FLAIR signaling, a hypointense aspect in T1 sequence, and maximum dimensions of 58/53/58 mm. The formation was accompanied by perilesional edema and caused a shift of the midline structures to the left by 22 mm. After the administration of gadolinium contrast, an inhomogeneous gadophilia aspect was observed. No other focal lesions or signal abnormalities were found intra-axially, infra, or supratentorially. The ventricular system was normal. There were no gyration abnormalities or signs of neuronal migration. Cerebral vascular axes were of normal diameter and conformation. Paranasal sinuses and mastoid cells were normally pneumatized. The patient underwent surgery in 2017, which involved the total resection of a right frontoparietal intracranial tumor. The histopathological examination results revealed a meningothelial meningioma. At discharge, the patient was conscious and cooperative, showing significant improvement in the initial neurological disturbances. There were no motor deficits, and the clinical aspect of the cranial nerves was normal. A follow-up CT examination performed in 2019 revealed the craniotomy site with a right fronto-temporo-parietal bony flap, a right frontal sequel hypodense area with a perilesional gliosis area, and a symmetrically located ventricular system with normal dimensions. The sagittal line structures were in a normal position. The patient presented at a neurological clinic in 2023 with bradypsychia, bradykinesia, progressive memory disorder, and left hemiparesis (MRC 4/5), along with a GCS score of 14 points. The patient denied experiencing headaches, vomiting, language disorders, or temporal-spatial disorientation. In this instance, the patient underwent a CT examination, which revealed voluminous intraparenchymal tumor formations with a hyperdense aspect. These were located at the right parieto-temporal level, with maximum dimensions of 73/33 mm, and at the fronto-basal level, with maximum dimensions of 92/58 mm. There was associated perilesional vasogenic edema with a significant mass effect, causing a 10 mm deviation of the midline structures, subfalciform herniation, and a tendency for uncal herniation. There were no recent ischemic lesions, no intraparenchymal, infra-, or supratentorial hemorrhagic accumulations, and a right fronto-parietal bony flap was observed.

**Figure 1:**
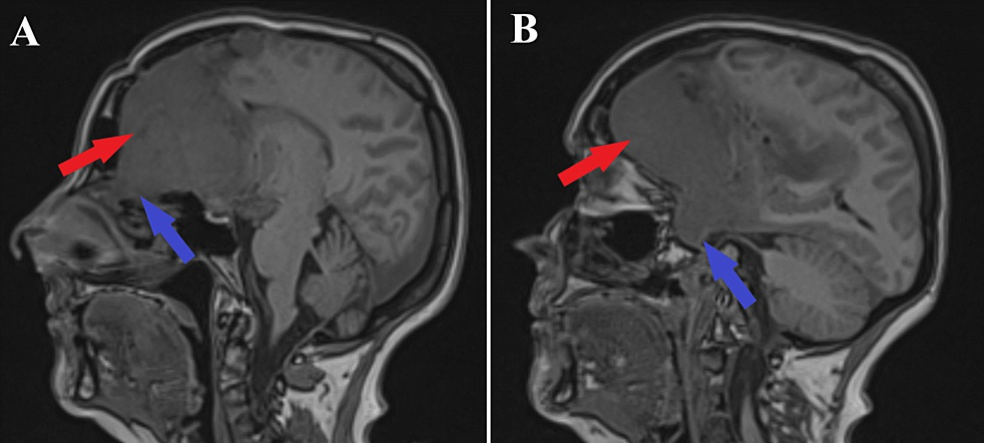
Preoperative MRI T1-sequence, sagittal section. Both images depict the tumoral process (indicated by red arrows) with significant orbital extension (A, shown by the blue arrow) and pituitary stalk implication (B, highlighted by the blue arrow).

**Figure 2:**
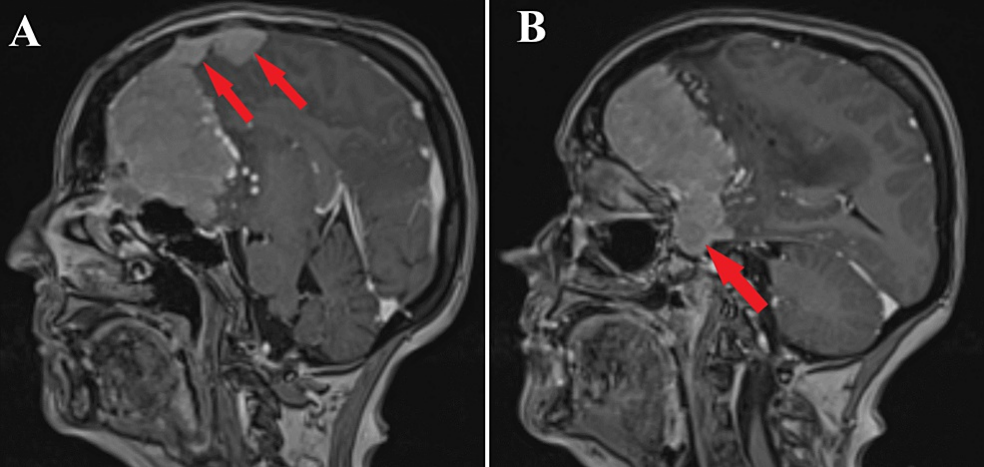
Preoperative MRI T1 post contrast sequence, sagittal section. A: Tumoral extension into the falx cerebri is represented (red arrows). B: Intrasellar and suprasellar tumoral segments are seen (red arrow).

**Figure 3:**
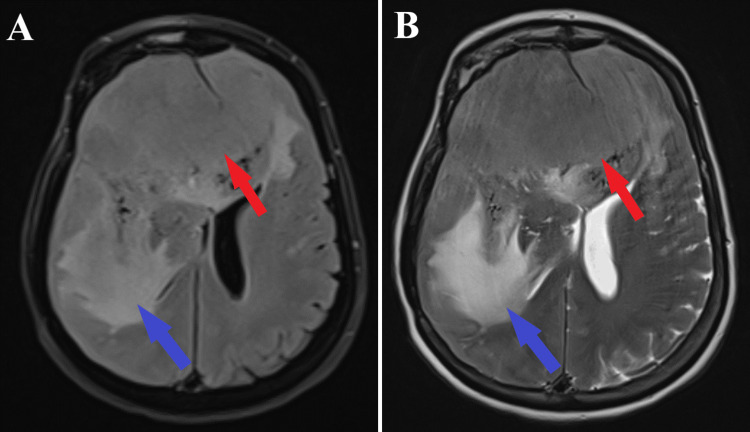
Preoperative MRI T2 and T2 FLAIR sequence, axial section. A: MRI T2 sequence showing the tumoral mass (red arrow) and significant perilesional edema (blue arrow). B: MRI T2 FLAIR sequence indicating the meningioma (red arrow) and the perilesional edema (blue arrow).

**Figure 4:**
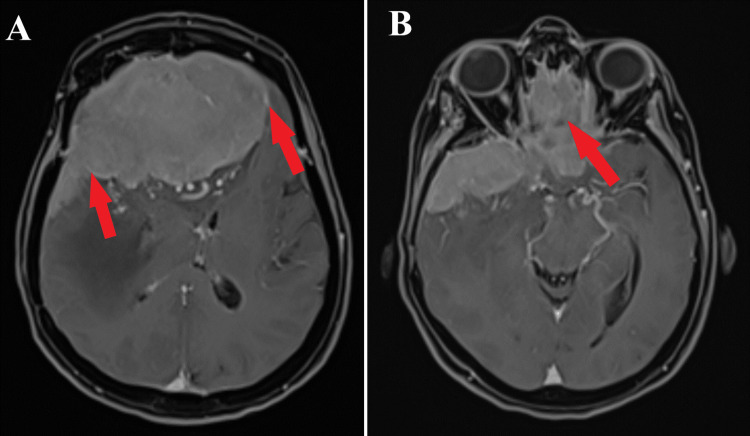
Preoperative MRI T1 post contrast sequence, axial section. A: Grasping post gadolinium administration is seen (red arrows). B: A significant orbitally located tumor is captured (red arrow).

**Figure 5:**
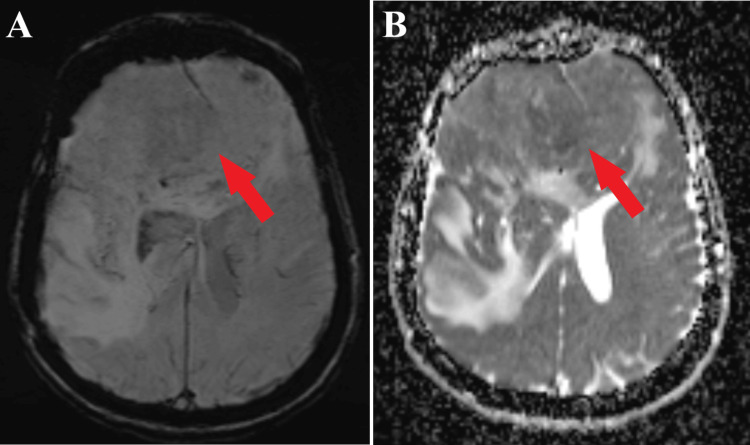
Preoperative MRI SWI and ADC sequence, axial section. A: MRI SWI sequence highlights the giant meningioma (red arrow). B: MRI ADC sequence depicts significant signaling within the meningioma (red arrow). SWI: Susceptibility Weighted Imaging; ADC: Apparent Diffusion Coefficient.

**Figure 6:**
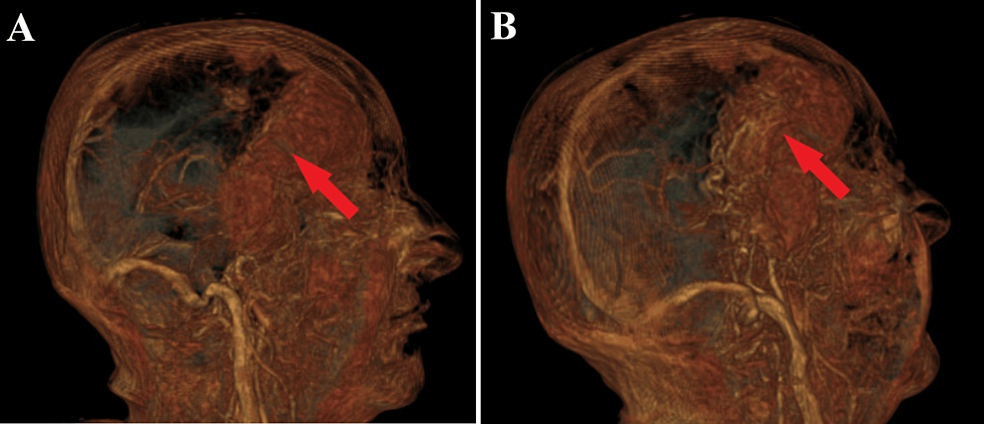
Preoperative 3D reconstruction. A: 3D reconstruction of MRI images depicts the giant meningioma in the axial plane (red arrow). B: 3D reconstruction of MRI images from a posterior viewpoint highlights the invasiveness of the meningioma (red arrow).

A subsequent CT scan performed in the same year revealed a dense, intensely iodophilic, homogeneous, and bulky tumor mass. Measuring approximately 106/70/80 mm, it was located bilaterally in the frontal area with a right parieto-temporal extension. This mass was associated with marked perilesional edema and a significant mass effect on the midline structures, resulting in leftward deviation and collapse of the lateral ventricles, more prominently on the right side. Additionally, another formation with the same characteristics, measuring 25/20/15 mm, was located in the right frontal, parasagittal area. The patient was admitted to our clinic with complaints of frontal lobe mental disorders, comitial seizures, and intracranial hypertension syndrome of medium intensity. Notably, over the last year, the patient experienced a rapidly progressive worsening of symptoms. Neurological examination revealed frontal ataxia and comitial seizures; osteo-tendinous reflexes were present bilaterally. Cranial nerves were clinically normal, and the GCS score was 14 points. An MRI scan of the native brain with paramagnetic substance showed a tumor formation on the anterior floor of the skull base with bifrontal development extending to the level of the tuberculum sellae. It had insertion at the level of the olfactory canal, jugum sphenoidale, and anterior clinoid bilaterally, and also extended into the left-sided middle fossa floor of the skull base, with dimensions of 12/7 cm. The tumor was moderately hypointense to the brain in T1-weighted sequences and moderately hyperintense in T2-weighted sequences (Figures 1-3), with intense enhancement after gadolinium diethylenetriamine pentaacetate (DTPA) administration, suggestive of a meningioma (Figure 4). The brain appeared normal in T1 and T2-weighted sections and after gadolinium DTPA administration. Other MRI sequences described the tumoral process and associated specifics (Figures 5-6).

**Figure 7:**
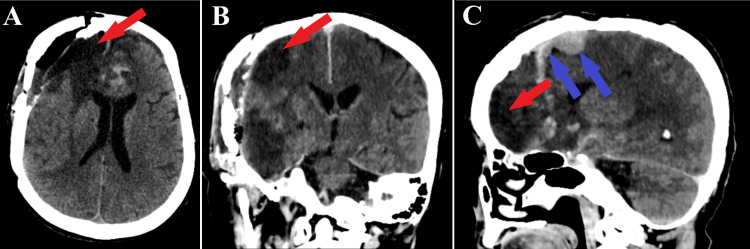
Postoperative CT scan. A: Axial section of the tissular CT window shows the complete tumor resection (red arrow). B: Frontal section of the tissular CT window highlights the total tumor ablation (red arrow). C: Sagittal section of the tissular CT window indicates the total resection of the frontal part of the tumor (red arrow) with a postoperative residual tumor fragment at the level of the falx cerebri (blue arrows).

Surgery was performed on the meningioma, and total tumor resection was achieved. A postoperative brain CT scan was conducted, which confirmed the complete resection of the meningioma. Additionally, a small tumoral remnant measuring 1.5/1 cm in size was observed at the level of the falx cerebri (Figure 7). Postoperatively, the patient showed a favorable evolution with improvement of frontal lobe mental disorders, resolution of comitial seizures, and remission of intracranial hypertension syndrome. The cranial nerves appeared normal during neurological examination, with a GCS score of 15 points at discharge.

## Discussion

Molecular research has revealed that numerous tumors, initially categorized as supratentorial embryonal based on histology, exhibit molecular profiles that align them with other tumor types, such as high-grade gliomas and ependymomas. This revelation holds significant therapeutic implications, particularly regarding the required radiotherapy volume for tumor control and the selection of adjuvant chemotherapy or biologic therapy [10]. In the context of central nervous system tumors, disease classification plays a pivotal role in dictating treatment strategies. Traditional risk assessment methods have depended on factors such as patient age, tumor histology, and metastatic status at diagnosis. While these factors serve as proxies for the complex underlying tumor biology, they do not fully account for the subtle molecular differences now recognized as crucial prognostic indicators [11]. Meningothelial (syncytial) meningiomas are the most prevalent histological type. The parasagittal region accounts for 24.22% of these tumors, followed by 19.95% at the sphenoid crest, 16.15% at the cerebral convexity, 15.67% at the anterior parafalcine, and 8.07% originating from the olfactory groove [12]. Meningothelial meningiomas comprise uniform tumor cells forming lobules encircled by collagenous septae. A characteristic feature of this subtype is the presence of clear spaces in the nucleus, indicative of absent karyoplasm. Unlike fibrous, psammomatous, or transitional meningiomas, whorls and psammoma bodies are less common and less distinct in meningothelial meningiomas. Notably, both meningothelial and fibrous meningiomas can influence the development and growth of adjacent gliomas [13].

Meningiomas extending from the meninges into extracranial tissue and causing skull osteolysis tend to follow an aggressive clinical trajectory. Surgical resection is advised for meningiomas demonstrating osteolytic reactions, extracranial extension, and a high MIB-1 index to prevent further aggressive growth [14]. These tumors are generally well-defined, unencapsulated, polypoid fibrous masses, exhibiting a spongy to solid tan-grey appearance upon dissection. They can vary significantly in size, reaching up to 22 cm, although the average size is around 4 cm. The composite of radiographic, clinical, and pathological staging ranges from tumors confined to the nasopharynx without bone destruction (stage I) to extensive invasion into the cranial cavity, cavernous sinuses, optic chiasm, or pituitary fossa (stage IV) [15]. Features such as a thickened pituitary stalk and invasion into adjacent structures like the cavernous sinus and the bony clivus may suggest a metastatic lesion. However, these can also manifest in other pituitary pathologies [16]. Despite advancements in understanding and treatment, skull base meningiomas present a persistent, albeit small, risk of recurrence, often with heightened clinical aggressiveness and biological characteristics. In rare cases, meningiomas originating from the sphenoid wings or middle cranial fossa can extensively infiltrate the craniofacial region, including the orbit, paranasal sinus, and nasopharynx, particularly upon recurrence through pathways such as the superior orbital fissure, pterygopalatine fossa, and infratemporal fossa [17]. Meningiomas in the paranasal sinuses may develop from heterotopic meningeal tissues, displaced during the closure of midline structures in fetal development, presenting symptoms like progressive exophthalmos, nasal obstruction, and epistaxis [18]. Although rare and typically benign, orbital meningiomas primarily arise from the sphenoid bone or optic nerve sheath. Anaplastic orbital meningiomas, which may originate from the frontal lobe, can be associated with orbital and distant extracranial metastases, especially post-aggressive frontal lobe meningioma resection. These meningiomas, accounting for 3-9% of all intraorbital neoplasms, are particularly noteworthy due to their location and potential to compress critical structures like the optic nerve, superior orbital fissure, cavernous sinus, and frontal and temporal lobes, posing significant surgical challenges [19,20].

The likelihood of recurrence and clinical behavior of these tumors are closely linked to their histological grade. The histological grade and the extent of surgical resection are primary predictors of recurrence. Higher-grade tumors, characterized by increased cellularity, a higher mitotic rate, and necrotic lesions, have a greater propensity for recurrence. The recurrence rates vary with WHO tumor grades, being 7%-25% for grade I, 29%-59% for grade II, and 60%-94% for grade III tumors [21]. Owing to their high recurrence rates, meningothelial meningiomas necessitate rigorous clinical monitoring and a tailored approach to treatment. Strategies such as re-operation, radiotherapy, or a combination of these therapies can be effective in managing disease progression while minimizing treatment-related adverse effects [22]. Cancer radiotherapy is a pivotal treatment modality aimed at destroying cancer cells through DNA damage induction. Ionizing radiation can cause both single-strand and double-strand breaks in the DNA double helix, which can be lethal if not rectified by the DNA damage response machinery. In the context of meningiomas unsuitable for surgical intervention, irradiation emerges as a viable option for controlling local growth. The application of adjuvant radiotherapy is established for grade III meningiomas, remains debated for grade II, and is generally not recommended for completely resected grade I meningiomas. Salvage radiotherapy is a practical choice for recurrent meningiomas, either as a standalone treatment or post-surgical excision [23]. The occurrence of meningiomas is noted in patients exposed to ionizing radiation, even at low doses such as those used for treating tinea capitis, with a heightened risk following high-dose radiotherapy. For instance, in children who have undergone cranial radiotherapy, the incidence of meningioma stands at 5.6% by the age of 40. Hormones, specifically cyproterone acetate, a synthetic steroidal antiandrogen, can amplify the risk of meningioma development by up to 11 times [24]. High-dose ionizing radiation is the sole recognized modifiable risk factor for meningioma development, with treatment warranted for symptomatic lesions or impending neurologic complications [25].

Gamma knife radiosurgery (GKS) is employed as an adjunct therapy following cranial surgery, offering precise high-dose irradiation with a sharp radiation profile. Despite its efficacy, instances of local control failure with GKS and tumor recurrence beyond the irradiation margins have been reported. The local control effectiveness of GKS post-cranial surgery for meningiomas has been assessed, but studies focusing on tumor progression, including recurrences outside the margin dose, are limited [26]. In cases where surgical intervention does not fully eradicate the lesion, adjuvant radiotherapy is also effective in preventing the recurrence of residual lesions. Nonetheless, meningiomas can recur adjacent to the irradiated site, with such recurrences often correlated with the histologic malignancy of the meningiomas [27]. GKS, particularly with a maximum dosage of 90 Gy directed at the trigeminal nerve, has been found to provide satisfactory long-term pain control, reduce medication usage, and enhance quality of life. However, higher dosage regimens may lead to increased sensory complications. The trade-off between higher dose benefits and risks, such as facial numbness, should be thoroughly discussed with patients, especially those experiencing severe pain [28]. The molecular classification of meningiomas facilitates the design of clinical trials, assigning patients to targeted treatments based on tumor genetic subtypes. The Hedgehog (Hh) signaling pathway, critical in embryogenesis and cell proliferation, is notably activated in some meningiomas, initiated by the binding of the Hh morphogen to its receptor, patched 1 (PTCH1) [29]. However, targeted therapies, particularly those addressing angiogenesis due to meningiomas' highly vascular nature and elevated proangiogenic factor expression, have yet to demonstrate significant efficacy [30].

## Conclusions

Our patient falls into the risk groups prone to strong recurrence of the pathology, exhibiting specific neurological dysfunctions such as gait apraxia, being of advanced age, and having the tumor located in areas known for a high risk of recurrence. Additionally, the presence of other pathologies may justify the increased incidence of recurrence. Our case demonstrates that current therapeutic tactics are not always sufficient for managing this type of pathology. Therefore, attention should be directed toward discovering new solutions to prevent the risk of recurrence or even the initial appearance of this tumor pathology.
